# Recurrent Cutaneous Squamous Cell Carcinoma with Direct Invasion of the Pleura: A Case Report

**DOI:** 10.7759/cureus.5115

**Published:** 2019-07-10

**Authors:** Akram Alkrekshi, Moneeb Mustafa, Hala Abdul-Al, Joanna Brell

**Affiliations:** 1 Internal Medicine, The MetroHealth System Campus of Case Western Reserve University, Cleveland, USA; 2 Pathology, The MetroHealth System Campus of Case Western Reserve University, Cleveland, USA; 3 Internal Medicine/ Hematology & Oncology, The MetroHealth System Campus of Case Western Reserve University, Cleveland, USA

**Keywords:** cutaneous squamous cell carcinoma, pleural effusion, direct pleural invasion

## Abstract

Cutaneous squamous cell carcinoma (cSCC) is the second most common skin cancer in the United States, second only to basal cell carcinoma. While majority of patients have a favorable outcome after surgical resection, a subset of patients carry a higher risk of local recurrence, distant metastasis, and mortality. In this article, we present an unusual case of a 54-year-old male who had trunk cSCC at the site of burn wound that recurred after surgical resection and radiotherapy. Interestingly the cSCC disease recurrence presented with respiratory symptoms secondary to malignant pleural effusion from direct invasion of pleura as the tumor eroded through the chest wall. The patient died within a few weeks from progressive disease. Despite the high incidence rate of cSCC, there is a paucity of randomized controlled trials to guide evidence-based management of cSCC in recurrent and metastatic disease.

## Introduction

Cutaneous squamous cell carcinoma (cSCC) is the second most common skin cancer in the United States, second only to basal cell carcinoma (BCC) [1]. Though the majority have a favorable prognosis after surgical resection, a subset of patients carry a higher risk of local recurrence, distant metastasis, and mortality [2]. Despite the high incidence rate of this malignant tumor, there is a paucity of randomized controlled trials (RCTs) to guide evidence-based management of cSCC, especially in recurrent and metastatic disease [3]. In this article, we present an unusual case of recurrent invasive trunk cSCC that eroded through the chest wall to the pleura resulting in malignant pleural effusion.

## Case presentation

54-year-old white male presented with three weeks of fatigue, dyspnea, and cough productive of white sputum. Past medical history was notable for burn injury at age 7 involving 90% total body surface, hypertension, hypertrophic cardiomyopathy, and mitral regurgitation. Nine months prior to this presentation, he sought medical care for 10 x 12 cm left flank burn scar ulcer, and multiple punch biopsies confirmed poorly differentiated cSCC. He was treated by surgical excision with 2 cm margin and split-thickness skin grafting. Lateral surgical margins were positive, and adjuvant radiotherapy of 6000 cGy in 30 fractions was delivered.

On examination, the patient was alert and oriented, afebrile, tachycardiac, tachypenic and requiring supplemental oxygen. Skin examination was notable for overt signs of burn scars throughout his whole body with significant scarring and decreased air-entry on the left lower chest. Cardiovascular, abdominal, and neurological examinations were normal. Laboratory tests were significant for leukocytosis and hypercalcemia of malignancy with suppressed parathyroid hormone levels.

**Figure 1:**
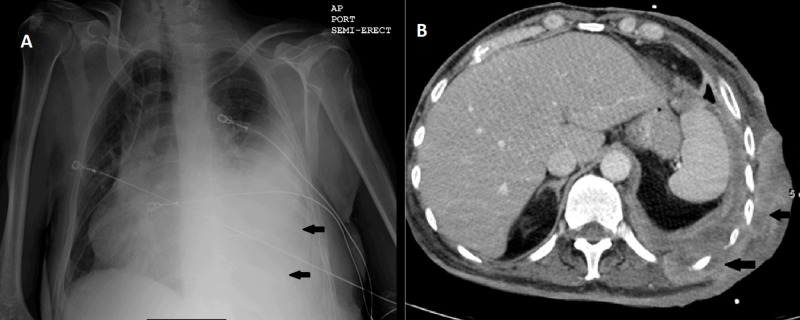
Chest X-ray and axial CT scan (A) Chest X-ray showing left-sided pleural effusion (arrows). (B) Axial CT image showing soft tissue mass invading the chest wall with pleural thickening and effusion (arrows).

Chest X-ray (CXR) showed left-sided pleural effusion (Figure 1A). Computed tomography (CT) of chest, abdomen, and pelvis showed marked thickening of left posterolateral chest wall skin with soft tissue extension, ribs destruction and bulging into left retroperitoneum and left lower pleura, large left-sided pleural effusion with pleural thickening, left lower lobe pneumonia, and new bilateral lung nodules concerning for metastasis (Figure 1B).

**Figure 2:**
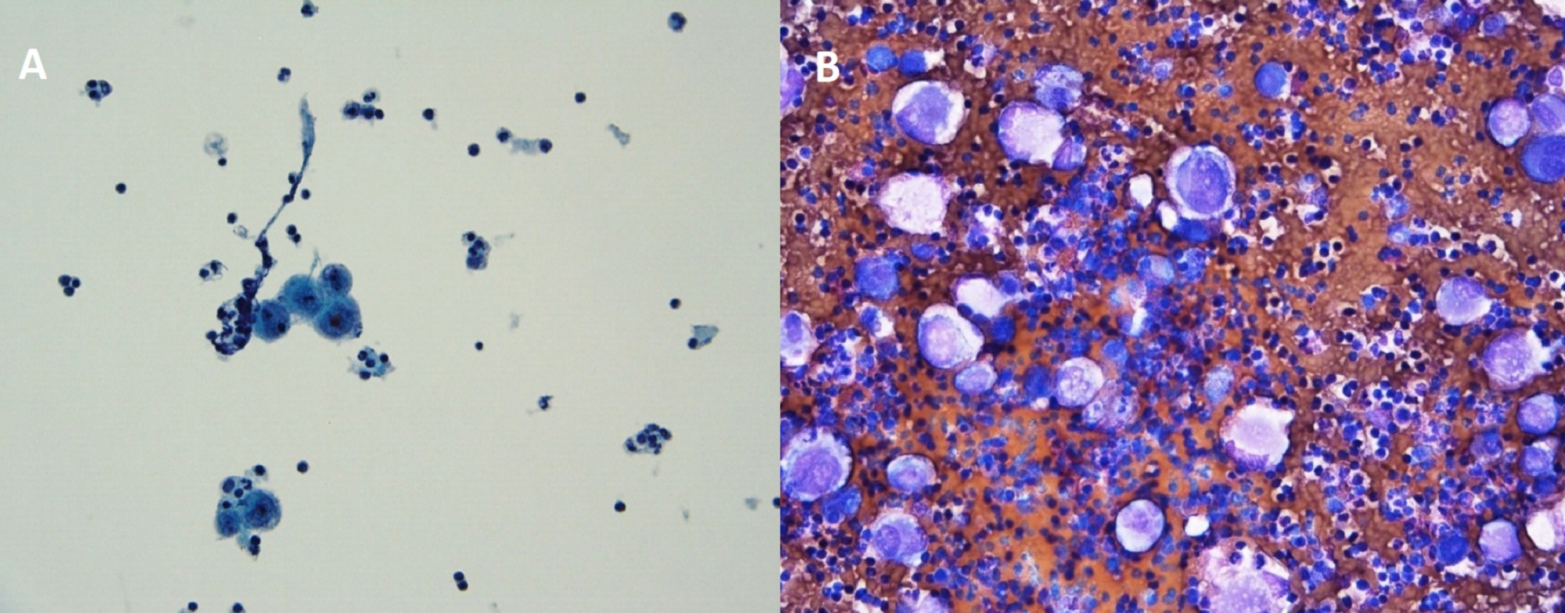
Cytology (A) Thin layer preparation, Papanicolaou stain showing malignant cells present in small clusters and singly in a background of lymphocytic effusion. The cells show a high nucleocytoplasmic ratio, prominent nucleoli and dense cytoplasm. (B) Cytocentrifuge preparation, Romanowsky-type stain showing malignant cells present in a background of lymphocytic effusion. The cells show high nucleocytoplasmic ratio, prominent nucleoli and variably dense cytoplasm.

**Figure 3:**
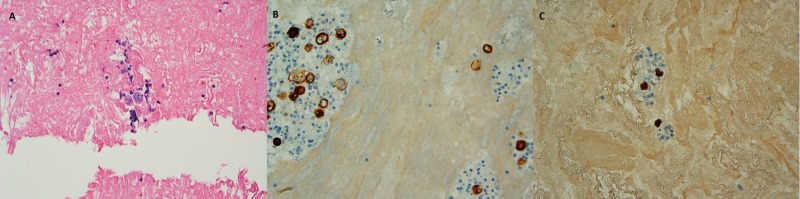
Cytology and immunohistochemistry (A) Haematoxylin and eosin stain showing poorly differentiated nonkeratinizing squamous cell carcinoma cells in cell block. (B) Squamous cell carcinoma cells show positive cytoplasmic staining for keratin by immunocytochemistry. (C) Squamous cell carcinoma cells show positive nuclear staining for P40 by immunocytochemistry.

Pleural fluid cytology showed malignant cells in small clusters and separately. The cells displayed a high nucleocytoplasmic ratio, prominent nucleoli and relatively dense cytoplasm (Figure 2). A battery of immunocytochemical stains was performed to further characterize the cells. The malignant cells were negative for TTF1, CK7, and B72.3 excluding a poorly differentiated adenocarcinoma. The cells were also negative for Melan A and Calretinin excluding malignant melanoma and mesothelioma, respectively. The malignant cells showed strong cytoplasmic immunoreactivity to keratin as well as strong nuclear staining with P40, diagnostic of poorly differentiated squamous cell carcinoma (Figure 3). The patient deteriorated in the following weeks and passed away from progressive disease.

## Discussion

Risk of local recurrence and distant metastasis increases when high-risk features are present: diameter of 2 cm or more, invasion of perineural or lymphovascular structures, deep extension beyond dermis, poor differentiation on histology, and cSCC occurring at the site of burn wound or chronic ulcer [4,5]. In such high-risk patients the chance of distant metastasis is comparable to renal cell and oropharyngeal carcinoma [2,5]. Management of local cSCC depends on the health status of the patient, the diameter of the lesion, and the presence of high-risk features. Low-risk cSCC that is less than 2 cm in diameter can be treated by curettage and electrodesiccation, cryotherapy or surgical excision. For high-risk cSCC Mohs micrographic surgery (MMS) is preferred. MMS is far superior when compared to tradition surgical excision with far lower recurrence rates, e.g. cSCC > 2 cm (25.2% versus 41.7%), in poorly differentiated cSCC (32.6% versus 53.6%), and cSCC with perineural invasion (0% versus 47%) [6].

Adjuvant radiotherapy of tumor site may be considered when there is surgical margin involvement, or when there is a high risk of recurrence. However, there are no RCTs that have studied the effectiveness of radiotherapy in trunk and extremities cSCC [6].

In a large RCT adjuvant chemoradiation using weekly carboplatin showed no added benefit when added to radiation in high-risk cSCC of the head and neck [7]. There are reports of tumor responses to various cytotoxic chemotherapies and EGFR inhibitors without enough evidence to high-level therapeutic recommendations [3]. Immunotherapy with cemiplimab (anti-PD-1), recently FDA approved for advanced and metastatic cSCC, has resulted in a response in about half of the patients [8]. Research for effective early detection techniques and novel therapies is necessary. Efforts should be directed towards further identification of driver mutations and actionable protein targets with the goal of decreasing morbidity and mortality from cSCC [9,10].

## Conclusions

This case demonstrates the aggressiveness of cSCC occurring in burn wound sites and the potential for localized disease recurrence to be undetectable prior to advanced symptoms, as in this patient who presented with respiratory symptoms. Imaging along with cytological studies of the pleural fluid confirmed direct invasion by cSCC to be etiology of pleural effusion. This is an exceedingly rare cause of pleural effusion and diagnosis can be challenging. Cutaneous squamous cell carcinoma is potentially lethal and further studies are needed to assess the role of adjuvant chemotherapy or immunotherapy (cemiplimab) to lower local recurrence rate or metastases rate in high-risk cSCC.
